# Testing the Sensitivity of Tract-Based Spatial Statistics to Simulated Treatment Effects in Preterm Neonates

**DOI:** 10.1371/journal.pone.0067706

**Published:** 2013-07-03

**Authors:** Gareth Ball, James P. Boardman, Tomoki Arichi, Nazakat Merchant, Daniel Rueckert, A. David Edwards, Serena J. Counsell

**Affiliations:** 1 Centre for the Developing Brain, Division of Imaging Sciences & Biomedical Engineering, King’s College London, London, United Kingdom; 2 MRC Centre for Reproductive Health, Queen’s Medical Research Institute, University of Edinburgh, Edinburgh, United Kingdom; 3 Biomedical Image Analysis Group, Department of Computing, Imperial College London, London, United Kingdom; University of Minnesota, United States of America

## Abstract

Early neuroimaging may provide a surrogate marker for brain development and outcome after preterm birth. Tract-Based Spatial Statistics (TBSS) is an advanced Diffusion Tensor Image (DTI) analysis technique that is sensitive to the effects of prematurity and may provide a quantitative marker for neuroprotection following perinatal brain injury or preterm birth. Here, we test the sensitivity of TBSS to detect diffuse microstructural differences in the developing white matter of preterm infants at term-equivalent age by modelling a ‘treatment’ effect as a global increase in fractional anisotropy (FA). As proof of concept we compare these simulations to a real effect of increasing age at scan. 3-Tesla, 15-direction diffusion tensor imaging (DTI) was acquired from 90 preterm infants at term-equivalent age. Datasets were randomly assigned to ‘treated’ or ‘untreated’ groups of increasing size and voxel-wise increases in FA were used to simulate global treatment effects of increasing magnitude in all ‘treated’ maps. ‘Treated’ and ‘untreated’ FA maps were compared using TBSS. Predictions from simulated data were then compared to exemplar TBSS group comparisons based on increasing postmenstrual age at scan. TBSS proved sensitive to global differences in FA within a clinically relevant range, even in relatively small group sizes, and simulated data were shown to predict well a true biological effect of increasing age on white matter development. These data confirm that TBSS is a sensitive tool for detecting global group-wise differences in FA in this population.

## Introduction

Preterm infants carry a profound risk of developing a spectrum of major disabilities and deficits in cognition, coordination and behaviour [1], [2] and microstructural alterations in the developing preterm white matter may underlie some of these deficits [3]–[5]. Magnetic resonance imaging (MRI) has been used extensively to study the developing brain, providing high-resolution images of the soft cerebral tissue without exposure to ionising radiation. Quantitative MR techniques have revealed that subtle, diffuse disturbances in the developing white matter may reflect early brain injury [6] and regional tissue loss, primarily in the cortical and subcortical grey matter, appears to accompany preterm white matter injury [7]–[9]. Converging evidence suggests that these changes predict neurocognitive and neuromotor abilities in early childhood and occur during a window of critical vulnerability in the neonatal period [10]–[13].

With increasing understanding of the biological pathways underlying preterm brain injury, early neuroprotective strategies for the preterm infant have been proposed and clinical trials undertaken with some success [14]–[16]. However, due to the relative time between treatment and clinical endpoint in such trials, they are limited in the speed and efficiency at which novel interventions can be evaluated. Without early markers of outcome, establishing surrogate biomarkers that are sensitive to preterm brain injury and predictive of future outcome is a pressing need for these children and essential for the planning and implementation of future clinical trials.

Neuroimaging is increasingly being employed to support biological and clinical research and for the discovery of potential biomarkers. We have previously shown that TBSS [17], a whole-brain DTI analysis technique, is sensitive to the effects of preterm birth, predictive of neurodevelopmental outcome and can be used as a powerful biomarker for neuroprotection in small groups of neonates with severe brain injury [5], [18]–[20]. TBSS may therefore represent a method for assessing the efficacy of future treatments strategies in preterm neonates in early phase trials, without the need to recruit large numbers of subjects or rely on long-term behavioural or functional follow-up assessments as clinical endpoints.

It is not clear how sensitive TBSS is to the subtle and diffuse alterations in the developing white matter that are known to predict functional outcome in preterm populations, particularly when sample sizes or effect sizes are relatively small. Previous studies have estimated that diffuse white matter injury in preterm infants can be associated with decreases in tissue anisotropy of up to 30% by term-equivalent age compared to healthy, term-born controls [18], [21], [22]. This suggests that an equivalent increase in a preterm population could predicate a clinically relevant recovery of function. Here, we model global, diffuse increases in fractional anisotropy (FA) of increasing size to test the ability of TBSS to detect such changes in the neonatal preterm brain. As proof of concept we compare these simulations to a true biological effect of increasing postmenstrual age at scan on white matter development.

## Materials and Methods

Ethical permission for this study was granted by the Hammersmith and Queen Charlotte’s and Chelsea (QCCH) Research Ethics Committee. Written parental consent was obtained for each infant.

### Subjects

DTI data acquired from 90 preterm infants (48 male) recruited from the Neonatal Intensive Care Unit at QCCH were used in this study. All infants were born at less than 36 weeks gestational age between March 2005 and October 2008. No infants had evidence of cystic periventricular leukomalacia or haemorrhagic parenchymal infarction at term-equivalent MRI. All were included in a previously reported TBSS study [19].

The cohort had a median (range) gestational age of 28^+6^ (23^+4^–35^+2^) weeks, median (range) birth weight of 1.13 (0.63–3.71) kg and a median (range) postmenstrual age at scan of 41^+4^ (38^+2^–44^+4^) weeks.

### Imaging

MRI was performed on a Philips 3-Tesla system (Philips Medical Systems, Netherlands) using an 8-channel phased array head coil. Single-shot EPI DTI was acquired in the transverse plane in 15 non-collinear directions using the following parameters: repetition time (TR): 8000 ms; echo time (TE): 49 ms; slice thickness: 2 mm; field of view: 224 mm; matrix: 128×128 (voxel size: 1.75×1.75×2 mm^3^); *b* value: 750 seconds/mm^2^; SENSE factor: 2.

All examinations were supervised by a paediatrician experienced in MRI procedures. Infants were sedated with oral chloral hydrate (25–50 mg/kg) prior to scanning and pulse oximetry, temperature and electrocardiography data were monitored throughout. Ear protection was used for each infant, comprising earplugs moulded from a silicone-based putty (President Putty, Coltene Whaledent, Mahwah, NJ) placed in the external ear and neonatal earmuffs (MiniMuffs, Natus Medical Inc, San Carlos, CA).

### Data Analysis

DTI analysis was performed using FMRIB's Diffusion Toolbox (FDT v2.0) as implemented in FMRIB's Software Library (FSL v4.1.5; www.fmrib.ox.ac.uk/fsl; [23]). Each infant’s diffusion weighted images were registered to their respective non-diffusion-weighted (*b* = 0) image to correct for spatial distortion due to eddy currents and subject motion. Images were brain extracted using Brain Extraction Tool (BET v2.1), diffusion tensors calculated voxelwise and fractional anisotropy maps calculated. TBSS [17] was performed using a modified pipeline specifically optimised for neonatal DTI analysis [19]. The aligned data were used to create a mean FA map and a mean FA skeleton that represents the centre of all white matter tracts common to the group. The FA skeleton was thresholded at FA ≥0.15 before each infant's aligned FA data were projected onto it.

### Simulating a Treatment Effect

**Figure 1:**
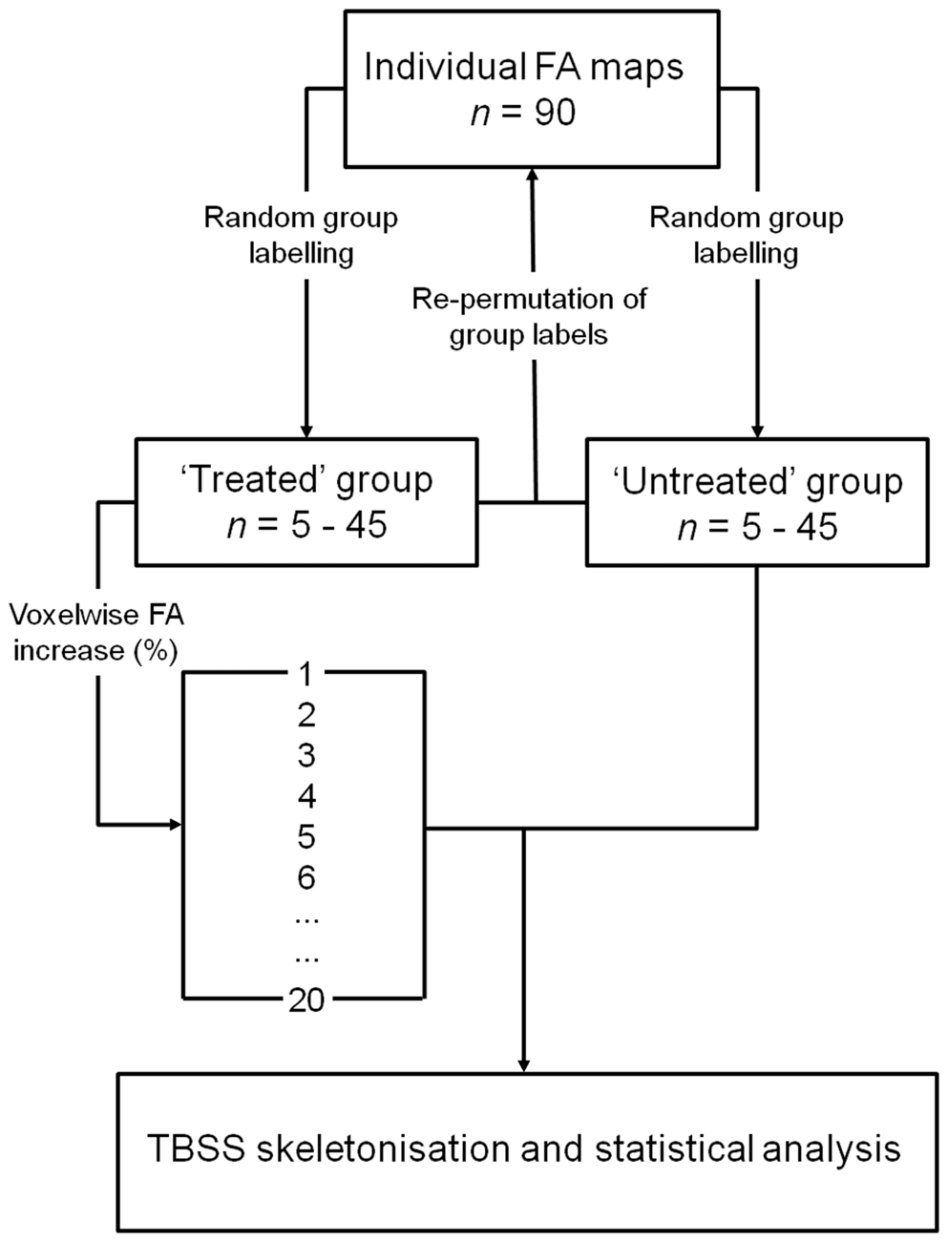
Processing pipeline to simulate global treatment effects in TBSS. FA maps were randomly assigned to ‘treated’ or ‘untreated’ groups. Voxel-wise increases in FA were used to simulate global treatment effects of increasing magnitude in all ‘treated’ maps. ‘Treated’ and ‘untreated’ FA maps were compared using TBSS.

Figure 1 illustrates the processing pipeline used to simulate global treatment effects. FA datasets were randomly split into ‘treated’ and ‘untreated’ groups of increasing size (5, 10, 15, 20, 25, 30, 35, 40 and 45 per group). For each group size, 20 treatment levels (and one unmodified control level) were created by increasing voxel-wise each ‘treated’ infant’s FA map by between 0 and 20% in 1% increments. At each treatment level, the artificially increased FA maps were aligned, along with the corresponding, unmodified FA maps from the ‘untreated’ group, to the TBSS skeleton using the previously calculated transformations and skeleton projections. To avoid bias due to the initial group composition, and to test the stability of the significant differences detected with TBSS, treatment labels were re-permuted a total of 10 times and TBSS repeated with each random group assignment. The mean proportion of significant skeletal voxels detected over all 10 permutations was used to estimate the sensitivity of TBSS to global increases in FA.

FSL’s Randomise (v2.5; www.fmrib.ox.ac.uk/fsl/) was used to perform a non-parametric, permutation-based statistical comparison of ‘treated’ and ‘untreated’ FA maps for each group size and at each treatment level. For each comparison, 1000 permutations were performed in Randomise to determine significance. In total, statistical analysis was performed 1890 times (10 group permutations×9 group sizes×21 treatment levels). After correcting for each infant’s gestational age at birth and age at scan, the number of voxels where a significant difference could be detected after each analysis was calculated as a percentage of the total number of voxels in the TBSS skeleton.

All images were subject to Family-Wise Error (FWE)-correction for multiple comparisons following threshold-free cluster enhancement (TFCE; [24]) and significant voxels were classified as p<0.05.

### Simulating Distributed Treatment Effects

**Figure 2:**
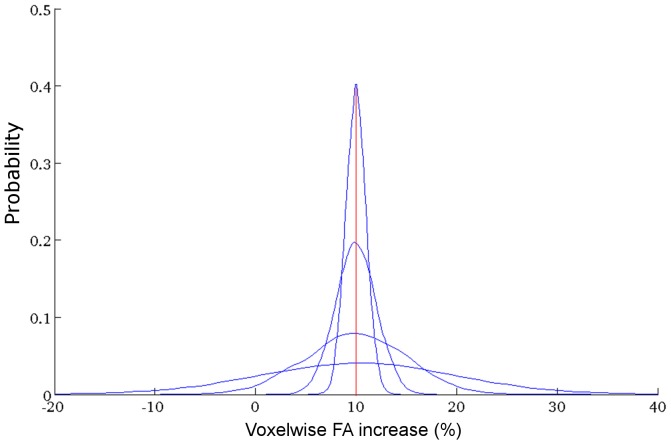
Modeling distributed treatment effects. Probability density functions of Gaussian distributions with mean effect size of 10% and increasing standard deviation (1%, 2%, 5% and 10%) are shown. Treatment effects were drawn from each distribution according to their probability. Distributions with means of 5%, 15% and 20% were also constructed but are not shown.

To more closely model clinical treatments, which are unlikely to produce alterations of equal magnitude in every subject, a further set of simulations was performed to test the sensitivity of TBSS when a range of treatment effects are simulated within a single ‘treated’ group. For a single set of groups of 20, 25, 30, 35, 40 and 45 infants in size, the magnitude of FA increase was drawn from a Gaussian distribution with mean of 5%, 10%, 15% or 20% and standard deviation of 1%, 2%, 5% or 10% (see Figure 2). Treatment effects were sampled with a likelihood given by the probability density function of the distribution and applied to ‘treated’ infants before TBSS was performed as described above. This process was repeated 10 times, each time drawing a new set of treatment effects from each distribution. A total of 960 TBSS analyses were performed (6 group sizes × 4 effect sizes × 4 distributions × 10 repeats).

### Validation

As proof of concept, the sensitivity of TBSS to simulated differences in FA was compared to the detection of age-related FA increases. Using TBSS, we have previously shown that increasing postmenstrual age at scan is significantly associated with global increases FA in preterm infants at term-equivalent age [19]. Here, FA maps were grouped according to each infant’s age at scan: older infants, scanned after 41 weeks, 3 days (*n* = 45), and younger infants scanned before then (*n* = 45). The proportion of significant skeletal voxels detected by TBSS between increasing group sizes of randomly selected old and young infants were compared to that predicted by the simulated data.

## Results

**Figure 3:**
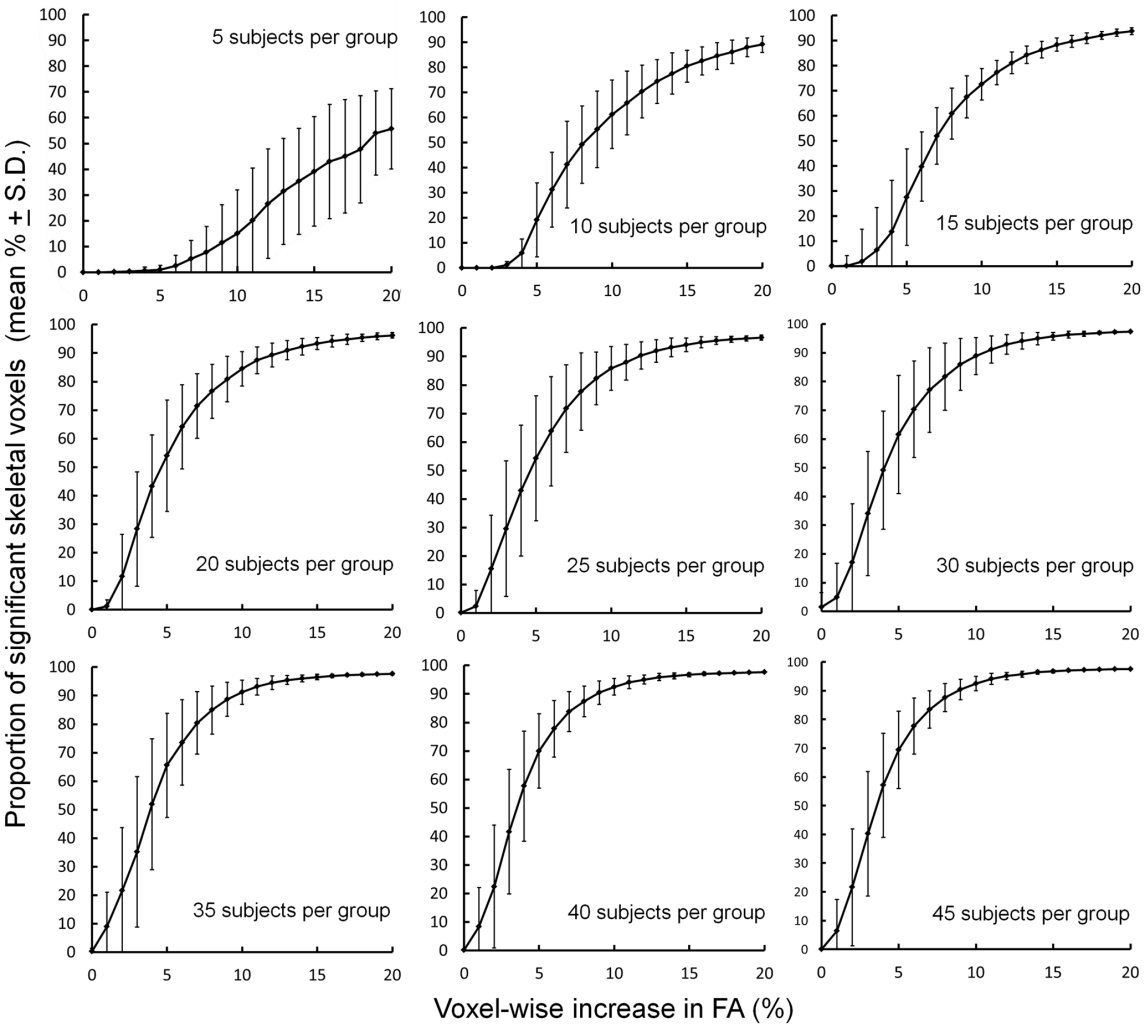
Detecting increasing simulated differences in FA between ‘treated’ and ‘untreated’ groups of increasing size using TBSS. Plots show the mean proportion of voxels across the mean FA skeleton where a significant difference (FWE-corrected, p<0.05) between ‘treated’ and ‘untreated’ groups could be detected.

The proportion of significant skeletal voxels detected between ‘treated’ and ‘untreated’ groups at each treatment level and for each group size are shown in Figure 3. Increasing the group size and simulated FA difference between groups resulted in a greater number of significant voxels detected across the FA skeleton. These data show that a mean increase in FA of around 5% is sufficient to detect a widespread ‘treatment’ effect (defined here as a visible effect in more than 50% of skeletal voxels) when comparing equal group sizes of 20 or more.

**Figure 4:**
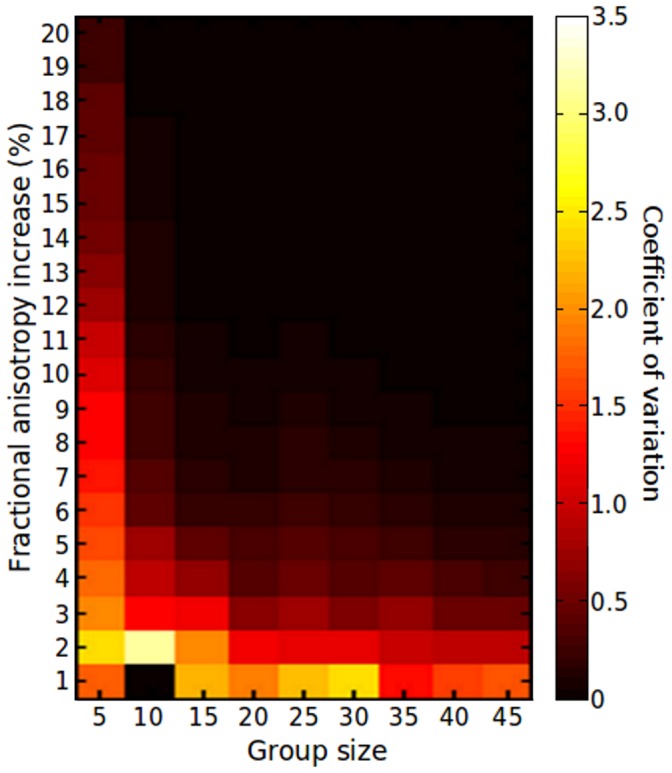
Variation in the detection of group differences between ‘treated’ and ‘untreated’ FA maps. The coefficient of variation calculated across 10 group permutations is shown in order to demonstrate the variance in the number of significant voxels detected by TBSS for each group size and treatment level.

To demonstrate the degree of variation in the number of significant voxels detected by TBSS, the coefficient of variation (C_v_) was calculated across each set of 10 permutations for every group size and treatment level. The C_v_ is a normalised measure of dispersion that shows the extent of variation in relation to the mean of a sample. Figure 4 shows that the C_v_ was substantially lower (i.e.: the number of significant voxels detected in each group permutation was closer to the estimated mean of all permutations) when TBSS was used to compare larger group sizes, or to detect larger simulated differences in FA. The increased variance observed in smaller group comparisons resulted in some global increases being artefactually represented as significant regional increases although few significant voxels were detected when comparing the control (i.e.: unmodified) treatment group with the untreated group. 9 out of 90 control comparisons resulted in a false positive result, although the number of voxels detected in each was small (Mean % significant voxels = 2.73%).

**Figure 5:**
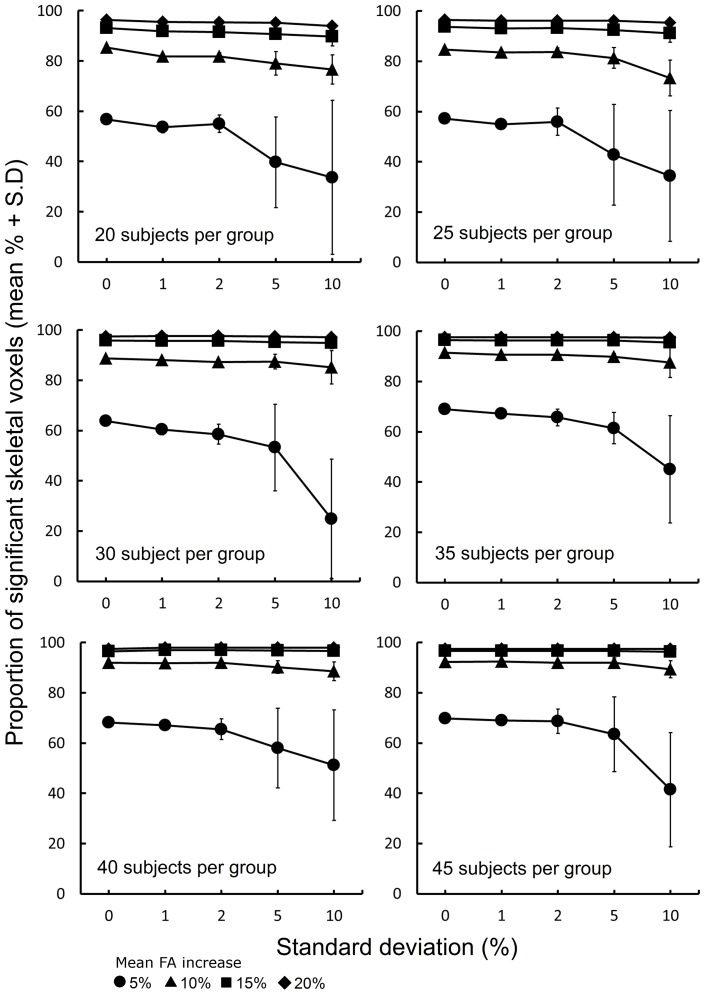
Sensitivity of TBSS to a distributed range of treatment effects. For group sizes between 20 and 45, plots show the proportion of skeletal voxels where a significant difference could be detected (FWE-corrected, p<0.05) between ‘treated’ and ‘untreated’ groups when treatment effects were drawn from Gaussian distributions with a mean of 5%, 10%, 15% and 20% FA increase and increasing standard deviation. The first point of each plot shows the % significant skeletal voxels detected when every ‘treated’ infant FA map was increased by exactly 5%, 10%, 15% or 20% voxelwise.

The effect of modelling a range of treatment effects within each treatment group is shown in Figure 5. TBSS sensitivity is most stable in larger treatment groups, or when a larger treatment effect is simulated. In general, when the standard deviation of the FA increase applied to ‘treated’ infants is less than 5%, the sensitivity of TBSS to detect significant differences approximates that observed after applying an equal treatment effect to all FA maps.

### Validation

**Figure 6:**
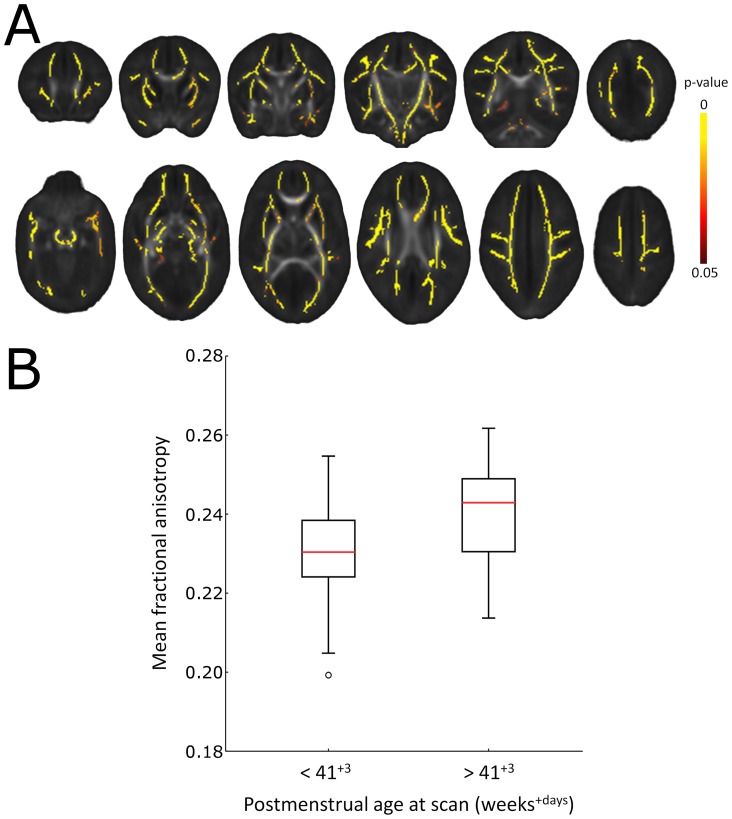
Global increases in FA according to postmenstrual age at scan. Significantly increased FA (yellow regions; p<0.05, FWE-corrected) in infants scanned at more that 41^+3^ days postmenstrual age (*n* = 45) compared to those scanned before then (*n* = 45) (A). FA values were extracted from across the whole TBSS skeleton and are plotted in B.

To validate these observations in a specific clinical comparison, simulated data were compared to the global effects of increasing postmenstrual age at scan on FA. As an exemplar, all infants were split into two equal sized groups according to their age at scan (before or after 41^+3^ weeks). TBSS comparison of old and young groups confirmed that increasing age was associated with a widespread increase in FA, detected across the mean skeleton (Figure 6A). Extracted FA values from the TBSS skeleton revealed a mean increase of 4.14% in the older group (Old group: mean FA ± S.D. = 0.240±0.013; Young group: 0.231±0.012; Figure 6B).

**Figure 7:**
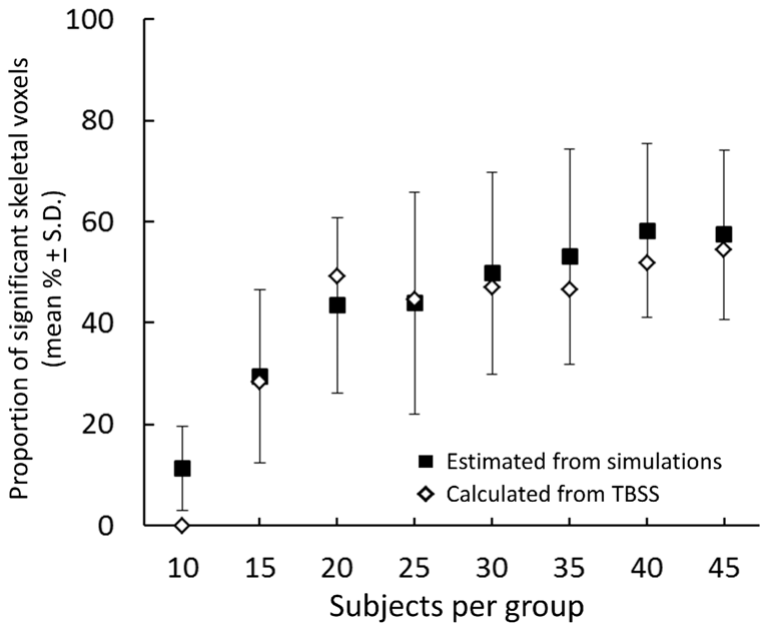
Comparison of simulated data with an age-based TBSS comparison. The proportion of significant voxels expected from the simulated data are plotted (Black squares; mean ± S.D.) alongside the actual proportion detected by exemplar TBSS analysis of ‘older’ and ‘younger’ infants.

Figure 7 shows the estimated mean proportion (± S.D.) of significant skeletal voxels expected from a 4.14% increase in all group sizes according to the simulated data (Figure 7; black squares). For comparison, the old and young groups were then randomly split to form smaller groups of 10 to 40 infants each. TBSS was performed once in each of these smaller groups and the number of significant skeletal voxels detected shown in Figure 7 (white diamonds). The simulated data predicts well the expected sensitivity of TBSS to a global biological effect when comparing groups of 25 or more.

## Discussion

We have shown by simulating treatment effects in a cohort of preterm infants that TBSS can readily be used in neonates to detect global differences in FA within a clinically relevant range, even in relatively small group sizes. We demonstrate that the simulated data can be used to estimate the sensitivity of TBSS to a biological effect of increasing age at scan on white matter development. These data indicate that using TBSS as a biomarker may allow early trials of potential neuroprotective strategies to measure outcome earlier in development and with fewer subjects. The data also provides a guide to estimate the sensitivity of TBSS for future study design in this population.

With the poor prognosis of many preterm infants in later life, much attention is focused on developing neuroprotective strategies to target the early neural substrates of neurodevelopmental deficits commonly seen in this population [25], [26]. In recent years, it has been shown that up to 75% of preterm infants display diffuse alterations in the developing white matter by term-equivalent age [27], alterations that have been quantified as changes to tissue microstructure that, in part, predict poor neurodevelopment [3], [5], [6], [28], [29]. Computational MR analysis has already proven powerful in investigating the efficacy of potential therapeutic strategies in neonates [20] and with the increasing availability of advanced imaging in the clinical setting, robust, objective and powerful image analysis techniques such as TBSS have the potential to aid clinical decisions and provide early biomarkers for future trials.

To demonstrate the potential sensitivity of TBSS in such trials, we simulated a systemic treatment effect by increasing FA across the whole brain by between 1 and 20%. The global pattern and treatment levels were selected to reflect the potential improvement putative neuroprotectants could provide and is in line with the magnitude and pattern of microstructural alterations previously observed in this population. We have previously shown with TBSS that prematurity at birth is associated with a global reduction of white matter FA at term-equivalent age [19]. These observations are consistent with region-of-interest studies that demonstrate reductions in FA of similar magnitudes across several white matter regions [21], [22]. Although few published trials of early interventions for preterm children using MRI to assess early brain development exist, systemic administration of neuroprotective agents such as melatonin in experimental models of preterm brain injury results in reduced white matter damage in several white matter structures including the corpus callosum, cingulate, external capsule and throughout the periventricular white matter [30], [31]. Additionally, we have shown that hypothermic treatment of neonatal brain injury results in global increases in FA that are predictive of favourable outcome in early childhood [20], [29] and two non-pharmacaological, randomized controlled trials that aimed to improve outcome in preterm infants by reducing stressful experiences during treatment in the NICU, found improvements in diffusion measures across several white matter regions-of-interest in the range of 5–15% [32], [33].

In terms of the magnitude of treatment effects, using TBSS in term-born infants with hypoxic ischemic encephalopathy, it was demonstrated that moderate hypothermia induced a mean (± S.D.) increase in FA of 31.3 (±7.8)% across the white matter [20]. In the absence of focal cerebral lesions, treatment effects in the majority of preterm infants are not likely to be as dramatic. However, early alterations in developmental care can result in increases in FA of around 15% that are associated with improved behavioural function in infancy and neurodevelopment in childhood [32], [34]. Furthermore, we have previously demonstrated that chronic lung disease, already shown to be independently associated with developmental outcome in preterm infants [35], is associated with a 6.4% decrease in FA across the majority of the FA skeleton [19]. Respiratory morbidity is an example of a potentially modifiable risk factor in this population that results in widespread decreases in the microstructural integrity of developing white matter, treatment of which may result in changes approximate to those modeled in this study. We propose that these data demonstrate the potential of TBSS in evaluating the short-term impact of putative neuroprotectants in future early-phase clinical trials.

These data could represent a guide to those planning a similar study in neonatal populations; however some limitations must be considered. Firstly, despite recent advances in the field, few software tools exist for power calculation in neuroimaging studies [36], [37]. Traditional power analysis techniques are not directly applicable to neuroimaging due to the complex nature of the data. Those techniques that do exist are designed for functional MRI (fMRI) analysis and depend on either *a priori* region-of-interest selection that doesn’t account for multiple comparisons between correlated voxels [36], or assumptions based on random field theory that are violated by TBSS and its use of non-parametric statistics to determine significance [37], [17], [24]. Although we are unable to provide a true power analysis, the work presented here is analogous to earlier attempts at defining power empirically in fMRI analysis [38], where groups of individual voxelwise activation maps obtained from fMRI were subsampled and repermuted and power defined as the % overlap between the resulting group maps and a gold standard (as shown in Figure 3).

Further it is unlikely that a real course of treatment would result in a response of equal magnitude in every individual. To explore this issue, we simulated a range of treatment effects at each level and demonstrated that the sensitivity of TBSS to detect global changes in FA is relatively stable across a range of treatment levels. Although this simulation better approximates the variation in treatment response that could be expected in a clinical trial, our relatively simple model of global change in FA does not account for possible regional variation in treatment response or non-linear treatment effects. Future work could further extend this framework to include a range of treatment levels and patterns based on earlier experimental pharmacokinetic or dose-ranging studies on a case-by-case basis.

We observed a false positive rate of 10%, in terms of significant differences detected between unmodified FA maps. This has some implication for the treatment analysis, and other TBSS analyses in that a number of significant regional ‘effects’ were detected in groups after a relatively small global increase in FA. This suggests that TBSS is highly sensitive to group composition, particularly in small group sizes when only supra-threshold voxels are considered as a measure of significance. Therefore, when a significant regional effect is observed between two relatively small groups without due cause, the possibility that it underlies a more global effect that the study is underpowered to detect should be considered. Note that this is likely to be true for all voxel-based, whole-brain analyses.

Finally, artificial noise was not added to the simulated treatment effects on a voxel-by-voxel basis. The inclusion of an arbitrary amount of Rician-distributed noise is often necessary when performing diagnostic tests with fully simulated MR data in an attempt to replicate the characteristics of a true MRI dataset [39]. In this study, treatment effects were based on a voxelwise increase of FA in MR datasets acquired *in vivo*, hence a realistic amount of noise was already present within the images tested, although noise characteristics can differ according to MR scanner and acquisition protocol.

### Conclusions

In summary, TBSS represents whole-brain, observer-independent DTI analysis methods shown to be sensitive to global increases in FA in a neonatal population. In addition, simulated DTI data can be used to estimate the sensitivity of TBSS to real biological effects. This suggests that TBSS represents a powerful tool in the search for early biomarkers of cerebral development, neurodevelopment outcome and neuroprotection in this vulnerable population.
